# Prevalence of Technology-Facilitated Abuse Among Sexual and Gender Minority Youths

**DOI:** 10.1001/jamanetworkopen.2023.54485

**Published:** 2024-02-02

**Authors:** Heather A. Turner, David Finkelhor, Kimberly Mitchell, Deirdre Colburn

**Affiliations:** 1 Crimes Against Children Research Center, University of New Hampshire, Durham, New Hampshire

## Abstract

**Importance:**

Digital communication and imaging technologies have created new opportunities for technology-facilitated abuse (TFA) and necessitate a better understanding of how and why the risk for TFA varies across different groups of youths.

**Objectives:**

To compare the prevalence of TFA among youths across 5 different sexual and gender identity groups, and to identify risk factors that may explain variations in TFA risk.

**Design, Setting, and Participants:**

A US nationally representative online survey was conducted among 2639 young adults recruited from the KnowledgePanel online panel from November 19 to December 29, 2021. Individuals aged 18 to 28 years were asked retrospectively about TFA and a variety of other youth experiences in childhood (aged <18 years).

**Main Outcomes and Measures:**

Participants were asked about their sexual and gender identities, 11 types of TFA occurring before the age of 18 years, and several potential risk factors. Prevalence rates of any TFA were compared across sexual and gender identity groups, and analyses were conducted to identify risk factors that help explain group differences. Weights were developed to compensate for gender, education, race and ethnicity, household income, census region, and metropolitan status disproportions.

**Results:**

Among the 2510 respondents (mean [SD] age, 24.8 [2.8] years) who provided gender and sexual identity information, 46.5% (95% CI, 41.9%-51.1%) were cisgender heterosexual females, 25.2% (95% CI, 21.6%-29.1%) were cisgender sexual minority females, 18.3% (95% CI, 14.4%-22.9%) were cisgender heterosexual males, 6.8% (95% CI, 4.6%-9.9%) were cisgender sexual minority males, and 3.3% (95% CI, 2.0%-5.4%) were gender minority individuals. The prevalence of TFA occurring before the age of 18 years was highest among sexual minority females (55.3%; 95% CI, 48.0%-62.4%) and gender minority individuals (53.4%; 95% CI, 33.5%-72.2%). Rates of sexting were highest among cisgender sexual minority females (38.8%; 95% CI, 19.1%-58.8%) and gender minority individuals (36.7%; 95% CI, 19.1%-58.8%) and represented the strongest risk factor for TFA exposure (odds ratio, 5.7; 95% CI, 4.0-8.0). However, it explained little variation in TFA across groups once early in-person adversity, sexual abuse, bullying, and maltreatment were taken into account.

**Conclusions and Relevance:**

This study demonstrates the risk of TFA among sexual and gender minority youths and the need to target prevention efforts toward these vulnerable groups. In addition to strategies to reduce risky behavior, efforts to lower the risk of early sexual abuse, bullying, and maltreatment are likely to also be effective in decreasing TFA among these marginalized groups.

## Key Points

**Question:**

Which groups of sexual and gender minority youths experience the highest prevalence of technology-facilitated abuse (TFA)?

**Findings:**

In this national survey study of 2510 young adults, cisgender sexual minority females and gender minority individuals experienced the highest rates of TFA before the age of 18 years (55.3% and 53.4%, respectively). Although sexting and risky online behavior were strong risk factors, early adversity, sexual abuse, bullying, and maltreatment explained the elevated risk among sexual and gender minority youths.

**Meaning:**

This study suggests that sexual and gender minority youths experience high rates of TFA and are important target populations for prevention and intervention efforts.

## Teaser

This survey study compares the prevalence of technology-facilitated abuse among youths across 5 different sexual and gender identity groups and identifies risk factors that may explain variations in technology-facilitated abuse risk.

## Introduction

The arrival of new digital communication and imaging technologies have created new opportunities for technology-facilitated abuse (TFA) of youths. Technology-facilitated abuse includes a variety of digitally based forms of abuse, including image-based sexual exploitation, such as nonconsensual sexting (ie, sharing a sexual image without permission), revenge pornography (ie, sharing a sexual image nonconsensually to hurt, humiliate, or get back at the person), and sextortion (ie, threatening to share a sexual image to get money, another image, or something else of value from the person). It also includes offenses such as online sexual solicitation (ie, unwanted requests for sexual interactions), commercial sexual exploitation (ie, exchange of sexual acts, images, or communications for money or something else of value), and cyberstalking (ie, unwanted contact via technology that communicates a threat and/or creates fear). A better understanding of how the risk and characteristics of TFA vary across different groups of youths would provide important information concerning how to target preventions and intervention efforts.

Research has found that youths who identify as sexual minorities (gay, lesbian, bisexual, pansexual) and/or who identify as gender minorities (transgender, gender fluid, other nonbinary gender) experience higher rates of discrimination, bullying, in-person sexual assault, and dating violence than do cisgender heterosexual youths.^1,2,3^ Turner et al^4^ found sexual minority status to also be a significant risk factor for online child sexual abuse, a category that includes several forms of TFA. Studies have also shown that sexual minority youths are more likely to have been pressured to send sexts and may be more susceptible to online grooming by adults.^5^ Although few studies have examined technology-facilitated sexual abuse specifically among gender minority individuals, one study of adults found that lifetime prevalence of digital harassment and abuse was significantly higher for transgender individuals relative to cisgender heterosexual participants.^6^

Although reasons for their elevated risk remain unclear, it has been suggested that sexual and gender minority (SGM) youths engage in more sexting behavior^7,8^ and more often use the internet for acquiring sexual information and finding sexual interactions,^9,10^ all of which can increase risk. Other possible risk factors include early puberty and early in-person exposure to various forms of abuse, bullying, and adversity that may be associated with subsequent online abuse.^4^

Although existing studies suggest an elevated risk of TFA among SGM youths as a whole, few studies have compared rates across specific gender and sexual identity groups, to our knowledge. Moreover, past studies have also been limited in the types of TFA assessed and their comprehensiveness of measurement. In addition, there is little information to date on how SGM youths may differ from non-SGM youths on risk factors that may help to explain their elevated rates of TFA.

The current study seeks to help fill these gaps by examining the prevalence and sources of risk associated with TFA before the age of 18 years in a nationally representative sample of young adults. Specific aims are to (1) compare the prevalence and characteristics of TFA experienced before the age of 18 years among 5 groups of young adults: cisgender heterosexual females, cisgender heterosexual males, cisgender sexual minority females, cisgender sexual minority males, and gender minority individuals; (2) identify differences in risk factors across the 5 sexual and gender identity groups, including sexual abuse, bullying, maltreatment, and adversity before the age of 13 years, early puberty, risky online behavior, and sexting; and (3) examine whether variations in the above risk factors help to explain group differences in TFA before the age of 18 years.

## Methods

The study was conducted using the nationally representative Ipsos online KnowledgePanel, a sample that the survey firm Ipsos has recruited via address-based sampling, gleaned from national universal address databases. After recruitment, participants agreed to participate in regular online surveys. Digital devices were provided to any sample members who lacked devices to participate. The KnowledgePanel panelists who were 18 to 28 years old were solicited for the current survey. In total, 2639 panel members participated in the survey, with a participation rate of 20%, and a response rate of 1%, accounting for original panel recruitment statistics.^11^ Such response rates are not atypical of modern survey research, and the KnowledgePanel design has been shown to be on par with what more traditional survey methods can currently provide.^12,13,14^ In addition to the consent agreement that Ipsos requires of all panel members, participants consented to the current online survey by clicking a “proceed” button after study-specific consent language. The study was approved by the human subjects review board of the University of New Hampshire. This study followed the American Association for Public Opinion Research (AAPOR) reporting guideline.

Of the 2639 completed surveys, 2510 provided gender and sexual identity information and 1215 answered 1 or more screening questions about TFA episodes. The sample was slightly older and more female compared with the US population of 18- to 28-year-old individuals. Weights were developed to compensate for age, gender, education, race and ethnicity, household income, census region, and metropolitan status disproportions and to adjust for nonresponse.

### Measures

#### Gender Identity and Sexual Orientation

Gender identity was assessed with the item: “Do you consider yourself?” With answers of (1) male, (2) female, (3) trans male, (4) trans female, (5) gender fluid or nonconforming, (6) don’t know, and (7) prefer not to answer. Sexual orientation was assessed with the question, “What is your sexual orientation?”, with response options including gay or lesbian, bisexual or pansexual, heterosexual, not listed (please specify), and prefer not to answer. From the above questions, 5 categories of sexual and gender identity were created: cisgender heterosexual male, cisgender heterosexual female, cisgender sexual minority male (male and gay, bisexual or pansexual, or other) cisgender sexual minority female (female and lesbian, bisexual or pansexual, or other), and gender minority (trans male, trans female, gender fluid or nonconforming, any sexual orientation).

#### Technology-Facilitated Abuse

In the current study, TFA is defined broadly to include many forms of online abuse and harassment. More specific categories of online offenses against minors are reported in an earlier publication.^15^ A measure was constructed representing any TFA if respondents answered yes to 1 or more of the following items, counting only incidents that occurred prior to the age of 18 years.

Nonconsensual image misuse: “Has someone ever shared with other people a sexual picture or video of you without your permission?”

Nonconsensual image taking: “Has someone ever taken or made a sexual picture or video of you without your permission?”

Forced image recruitment: “Has someone ever threatened, tried to force you, or strongly pressured you to provide sexual pictures or videos online or through a cell phone?”

Threatened sharing: “Has someone ever threatened to share a sexual picture or video of you to get you to do something—like take or send other sexual pictures of yourself, have a sexual relationship with them, pay them money, or something else?”

Unwanted sexual talk: “Did anyone ever use the internet or a cell phone to try to get you to talk about sex when you did not want to?”

Unwanted sexual questions: “Did anyone ever use the internet or a cell phone to ask you for sexual information about yourself when you did not want to answer those questions?”

Unwanted sexual acts requests: “Did anyone ever use the internet or a cell phone to ask you to do something sexual that you did not want to do?”

Voluntary older partner: “Did you have intimate sexual conversations or share sexual pictures or videos (online or through a cell phone), even if you wanted to, with a person who was 5 or more years older than you?”

Commercial talk, images, or other: “Have you done any of the following things over the internet or a cell phone (including texting) in exchange for money, drugs, or other valuable items?” (1) Sexual talk; (2) making, sending, or posting sexual pictures or videos of yourself; or (3) any other sexual activity.

Cyberstalking: “Has someone ever repeatedly contacted you online, on the phone, or in person when you did not want it, in a way that made you very afraid, anxious, or angry?” This was counted only if respondent indicated in a follow-up question that they were contacted through technology (cellphone, texts, online).

### Risk Factors

#### Adversities

The survey included 10 items measuring adversity under the age of 13 years (eg, bad accident or illness, parental incarceration, someone close attempting suicide). These items were summed, and the top decile were coded as having high adversity.

#### Bullying

Bullying comprised 3 items from the Juvenile Victimization Questionnaire,^16,17^ including verbal sexual harassment, physical intimidation, and emotional bullying. A binary variable was created to capture those who experienced at least 1 of these types of bullying before age 13 years.

#### Maltreatment

The survey used 3 items from the Juvenile Victimization Questionnaire to measure child maltreatment, including physical maltreatment, emotional maltreatment, and neglect. Respondents who experienced at least 1 of these types before the age of 13 years were coded as having experienced child maltreatment.

#### Early Puberty

Experiencing early puberty was assessed by asking respondents if they went through puberty before, at the same time, or after other children their age. A binary measure was created to capture those who reported going through puberty before other children their age.

#### Risky Online Behavior

Risky online behavior was measured using 4 items to assess respondents’ online behavior before the age of 18 years, including intentionally visiting an X-rated website, meeting face-to-face with someone they first met on the internet, pretending on the internet to be a different kind of person than they really were, and sending insulting messages to someone on the internet. These questions were answered on a scale from 1 (“never”) to 5 (“most everyday”). The 4 items were combined to measure cumulative online risk, and those scoring in the top decile were considered high risk.

#### Sexting

Sexting was measured using 1 item that asked frequency of sending sexual pictures or videos of oneself to another person before the age of 18 years. Respondents who gave a response other than “never” were coded as having sexted before the age of 18 years.

### Statistical Analysis

Data were analyzed using Stata/SE, version 17.0 (StataCorp LP). Survey weights were applied during analyses to obtain population prevalence estimates for the 5 sexual and gender identity groups. To assess whether there were significant differences across groups in prevalence and risk factors, 2-sided χ^2^ tests were conducted. Weighted hierarchical logistic regressions were conducted to assess whether risk factors help to explain differences in the prevalence of TFA across the 5 sexual and gender identity groups. Model 1 of the logistic regression includes sexual and gender identity; model 2 adds adversity, sexual abuse, bullying, and maltreatment; and model 3 adds early puberty, risky online behavior, and sexting behavior. *P* < .05 was considered statistically significant.

## Results

Among the 2510 respondents (mean [SD] age, 24.8 [2.8] years) who provided gender and sexual identity information, 46.5% (95% CI, 41.9%-51.1%) were cisgender heterosexual females, 25.2% (95% CI, 21.6%-29.1%) were cisgender sexual minority females, 18.3% (95% CI, 14.4%-22.9%) were cisgender heterosexual males, 6.8% (95% CI, 4.6%-9.9%) were cisgender sexual minority males, and 3.3% (95% CI, 2.0%-5.4%) were gender minority individuals. Table 1 shows full sample characteristics, including the demographic distribution and variable statistics. Table 2 presents prevalence rates of TFA before the age of 18 years across the 5 sexual and gender identity groups. Substantial and statistically significant differences were evident (χ^2^=281.586; *P* < .001). Cisgender sexual minority females had the highest rate of TFA (55.3%; 95% CI, 48.0%-62.4%), similar to rates among gender minority individuals (53.4%; 95% CI, 33.5%-72.2%). The next highest prevalence of TFA was among cisgender heterosexual females (36.9%; 95% CI, 32.8%-41.1%), followed by cisgender sexual minority males (25.1%; 95% CI, 16.6%-36.0%), with cisgender heterosexual males having the lowest prevalence of TFA during childhood (12.9%; 95% CI, 9.9%-16.8%).

**Table 1.** Study Sample Characteristics

Characteristic	Weighted % (95% CI) (N = 2510)
Age, mean (SD), y	24.8 (2.8)
Gender and sexual orientation	
Cisgender heterosexual male	18.3 (14.4-22.9)
Cisgender heterosexual female	46.5 (41.9-51.1)
Cisgender sexual minority male	6.8 (4.6-9.9)
Cisgender sexual minority female	25.2 (21.6-29.1)
Gender minority youth	3.3 (2.0-5.4)
Technology-facilitated abuse	
Any	28.9 (26.4-31.6)
Adversity	
High before age of 13 y	10.0 (8.4-11.9)
Bullying	
Any before age of 13 y	20.4 (18.3-22.6)
Maltreatment	
Any before age of 13 y	16.8 (14.9-18.9)
Early puberty	
Yes	9.4 (7.9-11.1)
Risky online behavior	
High before age of 18 y	9.9 (8.3-11.7)
Sexting	
Any before age of 18 y	20.8 (18.6-23.2)

**Table 2.** Prevalence of Any TFA Before the Age of 18 Years by Sexual and Gender Identity Group

Group	Any TFA <18 y, weighted % (SE) [95% CI] (N = 2510)^a^
Cisgender heterosexual male (n = 649)	12.9 (1.8) [9.9-16.8]
Cisgender heterosexual female (n = 1260)	36.9 (2.1) [32.8-41.1]
Cisgender sexual minority male (n = 127)	25.1 (5.0) [16.6-36.0]
Cisgender sexual minority female (n = 417)	55.3 (3.7) [48.0-62.4]
Gender minority (n = 57)	53.4 (10.4) [33.5-72.2]

Abbreviation: TFA, technology-facilitated abuse.

^a^ χ^2^ = 281.586; *P* < .001.

Table 3 presents differences in the hypothesized risk factors across the 5 sexual and gender identity groups. All factors were significantly different across the groups. High levels of early adversity and all forms of early in-person abuse and exploitation were least prevalent among cisgender heterosexual males followed by cisgender heterosexual females. Specifically, high adversity was highest among gender minority individuals (28.1%; 95% CI, 14.8%-46.4%; *P* < .001), followed by cisgender sexual minority males (20.0%; 95% CI, 11.9%-31.6%; *P* < .001). Early sexual abuse (before age 13 years) was most prevalent among cisgender sexual minority females (19.2%; 95% CI, 14.2%-25.4%; *P* < .001) and gender minority individuals (18.5%; 95% CI, 7.6%-38.3%; *P* < .001). The percentage of individuals experiencing early bullying and maltreatment was especially high among gender minority individuals (56.6%; 95% CI, 35.3%-75.7%; *P* < .001 and 42.4%; 95% CI, 25.1%-61.8%; *P* < .001, respectively) followed by cisgender sexual minority females (36.6%; 95% CI, 30.1%-43.6%; *P* < .001 and 32.0%; 95% CI, 25.9%-38.8%; *P* < .001, respectively). Both cisgender sexual minority females and gender minority individuals were substantially more likely than the other groups to report entering puberty earlier than their peers. Risky online behavior was most common among gender minority individuals (29.0%; 95% CI, 14.4%-49.8%; *P* < .001) and sexual minority cisgender males (24.4%; 95% CI, 15.7%-36.0%; *P* < .001) and least common among cisgender heterosexual females (7.2%; 95% CI, 5.4%-9.4%; *P* < .001). Cisgender sexual minority females and gender minority individuals had the highest rate of participating in any sexting before the age of 18 years (38.8%; 95% CI, 19.1%-58.8%; *P* < .001; and 36.7%; 95% CI, 19.1%-58.8%; *P* < .001, respectively), while cisgender heterosexual males had the lowest rate (11.9%; 95% CI, 9.2%-15.4%; *P* < .001).

**Table 3.** Risk Factors by Sexual and Gender Identity Category

Risk factor	Weighted % (SE) [95% CI] (N = 2510)				
	Cisgender heterosexual		Cisgender sexual minority		Gender minority (n = 57)
	Male (n = 649)	Female (n = 1260)	Male (n = 127)	Female (n = 417)	
Adversity (high)^a^	7.2 (1.5) [4.8-10.7]	8.1 (1.0) [6.3-10.3]	20.0 (5.0) [11.9-31.6]	16.3 (2.3) [12.2-21.4]	28.1 (8.3) [14.8-46.4]
Any sexual abuse^a^	3.3 (1.1) [1.7-6.1]	10.8 (1.3) [8.6-13.5]	12.1 (4.0) [6.2-22.2]	19.2 (2.8) [14.2-25.4]	18.5 (7.8) [7.6-38.3]
Any bullying^a^	11.3 (1.6) [8.6-14.7]	21.8 (1.7) [18.8-25.4]	25.7 (5.1) [17.0-36.8]	36.6 (3.5) [30.1-43.6]	56.6 (10.9) [35.3-75.7]
Any maltreatment^a^	10.3 (1.5) [7.8-13.5]	16.7 (1.6) [13.9-20.0]	20.0 (4.8) [12.2-31.1]	32.0 (3.3) [25.9-38.8]	42.4 (9.8) [25.1-61.8]
Early puberty	4.6 (1.1) [2.8-7.4]	11.8 (1.3) [9.4-14.7]	6.6 (2.4) [9.4-14.7]	17.2 (2.8) [12.4-23.3]	22.0 (8.5) [9.7-42.6]
Risky online behavior (high)	7.9 (1.3) [5.7-10.9]	7.2 (1.0) [5.4-9.4]	24.4 (5.2) [15.7-36.0]	13.2 (2.4) [9.2-18.6]	29.0 (9.3) [14.4-49.8]
Sexting (any)	11.9 (1.6) [9.2-15.4]	22.8 (1.8) [19.5-26.5]	24.9 (5.1) [16.2-36.2]	38.8 (10.7) [19.1-58.8]	36.7 (10.7) [19.1-58.8]

^a^ Includes only episodes that occurred before the age of 13 years.

Table 4 presents associations between the risk factors and exposure to TFA. Model 1 includes the 5 sexual and gender identity groups. Compared with heterosexual cisgender males, the odds of TFA were significantly higher in each of the other groups, consistent with their relative prevalence rates. In model 2, high adversity and the 3 in-person abuse types were added to the equation. All factors were associated with significantly increased odds of TFA. With early adversity, sexual abuse, bullying, and maltreatment accounted for, the elevated risk of TFA among both nonheterosexual males and gender minority individuals was reduced to nonsignificance and the risk among sexual minority females was reduced by 30% but remained substantially greater than for heterosexual males. In Model 3, early puberty, online risky behavior, and sexting were added to the equation. Both risky online behavior (odds ratio, 2.5; 95% CI, 1.5-4.2) and sexting (odds ratio, 5.7; 95% CI, 4.0-8.0) were associated with TFA, while early puberty had no independent association with TFA. Although, with these factors controlled, there was a minor additional reduction in the odds of TFA exposure among sexual minority females, the coefficient remained substantial and significant. The odds ratio for heterosexual females remained unchanged.

**Table 4.** Logistic Regression Analyses of the Odds of Any TFA Before the Age of 18 Years on Sexual and Gender Identity and Other Risk Factors

Risk factor	OR (SE) [95% CI] (N = 2510)		
	Model 1	Model 2	Model 3
Sexual and gender identity (reference: cisgender heterosexual male)			
Cisgender heterosexual female	3.9 (0.7) [2.8-5.6]	3.5 (0.7) [2.4-5.1]	3.6 (0.8) [2.3-5.4]
Cisgender sexual minority male	2.3 (0.7) [1.2-4.1]	1.5 (0.5) [0.8-2.7]	1.1 (0.4) [0.6-2.2]
Cisgender sexual minority female	8.3 (1.8) [5.4-12.7]	5.9 (1.3) [3.8-9.1]	5.2 (1.4) [3.1-8.7]
Gender minority	7.7 (3.4) [3.2-18.5]	3.5 (2.3) [1.0-12.4]	2.8 (1.4) [1.0-7.7]
Adversities (reference: not high)			
High	NA	2.0 (0.5) [1.2-3.4]	2.0 (0.6) [1.1-3.6]
Sexual abuse (reference: none)			
Any	NA	2.9 (0.9) [1.6-5.3]	2.2 (0.7) [1.1-4.2]
Bullying (reference: none)			
Any	NA	2.4 (0.4) [1.7-3.4]	2.1 (0.4) [1.4-3.2]
Maltreatment (reference: none)			
Any	NA	2.3 (0.5) [1.5-3.4]	1.8 (0.4) [1.1-2.8]
Puberty (reference: not early)			
Before other kids the participant’s age	NA	NA	1.2 (0.3) [0.8-2.0]
Risky online behavior (reference: not high risk)			
High risk	NA	NA	2.5 (0.6) [1.5-4.2]
Sexting (reference: never)			
Any	NA	NA	5.7 (1.0) [4.0-8.0]

Abbreviations: NA, not applicable; OR, odds ratio; TFA, technology-facilitated abuse.

## Discussion

The current study addresses important gaps in the literature by using a comprehensive assessment of TFA and using a nationally representative sample to estimate and compare TFA exposure across specific gender and sexual minority groups. Findings show that the prevalence of TFA occurring before the age of 18 years was high among SGM groups, with between 2 and 8 times the odds of exposure relative to heterosexual males. A clear pattern emerged: among cisgender individuals, females had higher rates than males, regardless of sexual orientation and, within genders, sexual minority individuals had higher rates than heterosexuals. As a result, a double disadvantage is evident in the high rates of TFA among sexual minority cisgender females. Gender minority individuals of any sexual orientation were also substantially disadvantaged.

All the hypothesized risk factors for TFA were significantly different across the 5 groups, with the relative differences largely mirroring variations in prevalence rates. Consistent with other research,^7,8^ findings showed that a significantly greater percentage of SGM individuals than cisgender heterosexuals had sexted and engaged in risky online behavior. However, multivariable analyses indicated that the higher rate of TFA among sexual minority males and, especially, gender minority individuals appeared to be largely explained by exposure to early (before age 13 years) adversity, sexual abuse, bullying, and maltreatment. These factors also explained part of the higher risk among sexual minority females. Thus, elevated abuse and exploitation among SGM youths appear to be established prior to adolescence, before the developmental stage in which technology-facilitated harassment and abuse become most prevalent.^15^ Although risky online behavior and sexting are potent risk factors for TFA, it is not their disproportionate use that explains the elevated risk among sexual and gender minority individuals. Instead, these youths appear to be vulnerable targets of abuse from a young age, abuse that moves from in-person bullying, maltreatment, and sexual abuse to later include online sexual abuse. Given strong linkages often found between online and offline forms of abuse and exploitation,^18,19^ it is plausible that TFA may also increase risk for subsequent in-person incidents. These finding suggest that SGM youths are likely at elevated risk not only for TFA but for what has been termed *polyvictimization* (experiencing multiple different types of abuse and exploitation), which has been found to substantially increase the risk of trauma symptoms.^20^

Elevated risk of TFA among cisgender heterosexual females, the largest group of survivors, was not explained by any of the risk factors considered in this study. Risk of TFA also remained substantial for sexual minority females with the risk factors controlled. Greater TFA evident among female youths reflects broader gender patterns in sexual offenses and the gendered power structure that underlies these behaviors. Boys can gain respect, popularity, and confirmation of masculinity through the possession and distribution of girls’ sexual images and proving their ability to gain access to girls’ bodies.^21,22^ Educational programs that address harmful gender-based norms around sexuality and encourage the development of healthy sexual relationships are likely to also be effective in reducing TFA risk among girls.

### Limitations

There are important limitations to this study. The incidents being analyzed were retrospective accounts, some more than 10 years old. In the rapidly changing digital world, they may not reflect current reality for young people. Although we attempted to establish temporal ordering of some risk factors for TFA, such as adversity and forms of in-person abuse, by counting only events that occurred prior to age 13 years, the causal order of variables remains somewhat ambiguous. Next, like most contemporary surveys, the overall participation rate was low. However, the probability-based sample, efforts to reduce sample bias, and weighting strategies increase our confidence in the accuracy of our results. Finally, sexual and gender identity were measured at the time of the study when participants were young adults. Given the fluidity of sexual and gender identities,^23,24,25^ it is possible the participants might have identified differently during the time frame of the experiences asked about in the current study.

## Conclusions

This study demonstrates the risk of TFA among SGM youths and the need to target prevention and intervention efforts toward these vulnerable youths. Because sexting and risky online behavior are strong proximal factors that increase the likelihood of TFA, our findings confirm the importance of developing prevention strategies, directed at all youths, to reduce these behaviors or to make them less risky. Education programs and online resources that provide supportive, nonjudgmental information and guidance on how to seek help and reduce risk online would be beneficial in this regard. However, this study also suggests that the important underlying factors associated with elevated exposure to TFA among SGM adolescents are early in-person adversity, bullying, and abuse. As such, TFA appears to be a component of the broader stigma and discrimination processes that contribute to stressful life conditions for SGM youths.^26^ Effective prevention programs directed at reducing risk of early sexual abuse, bullying, and maltreatment are likely to also be effective in reducing TFA among these marginalized youths.

## Notes

Supplement.
Data Sharing Statement
