# Supplementary material for: Prevalence of Technology-Facilitated Abuse Among Sexual and Gender Minority Youths
Source: JAMA Netw Open. 2024 Feb 2;7(2):e2354485. doi: 10.1001/jamanetworkopen.2023.54485 (PMC10837746; doi:10.1001/jamanetworkopen.2023.54485)
Supplement: Supplement. — Data Sharing Statement [file jamanetwopen-e2354485-s001.pdf]

Data Sharing Statement

Turner. Prevalence of Technology-Facilitated Abuse Among Sexual and Gender Minority Youths. *JAMA Netw Open*. Published February 02, 2024.
doi:10.1001/jamanetworkopen.2023.54485

Data
Data available: Yes
Data types: Other (please specify)
Additional Information: Data available upon request from Author
How to access data: Data available upon request from Author
When available: With publication

Supporting Documents
Document types: None

Additional Information
Who can access the data: Data will be made available to anyone through a publicly available data archived
Types of analyses: Data will be made available to anyone through a publicly available data archived
Mechanisms of data availability: Data will be made available to anyone through a publicly available data archived
Any additional restrictions: Data will be made available to anyone through a publicly available data archived
